# ‘Good’ and ‘bad’ doctors - a qualitative study of the Austrian public on the elements of professional medical identity

**DOI:** 10.1080/10872981.2022.2114133

**Published:** 2022-08-24

**Authors:** Julia S. Grundnig, Verena Steiner-Hofbauer, Henri Katz, Anita Holzinger

**Affiliations:** Teaching Center, Medical University Vienna, Vienna, Austria

**Keywords:** Medical professionalism, good doctor, qualitative study, professional identity formation, public involvement

## Abstract

Professional identity formation has become a key focus for medical education, but there is still much to learn about how to help students develop their professional identity. At a time when influential concepts such as public- and patient-centered care have become common values, there is little research on the conceptions of the public that trainees might adopt during their training. Defining characteristics of ‘good’ and ‘bad’ physicians can be a starting point when considering how to incorporate aspects of professional behavior into medical curricula. Therefore, this study examined the essential elements of physician identity from the public’s perspective. This study aimed to describe the Austrian public’s viewpoint about the characteristics of ‘good’ and ‘bad’ doctors. Using a qualitative research design, interviews were conducted with the Austrian public (n = 1000, mean age 46.4 ± 15.8 years). Interviews were transcribed verbatim and analyzed via qualitative content analysis. The respondents stated 2078 answers for ‘good’ and 1728 for ‘bad’ doctors. The content analysis produced seven categories: ‘social skills’ (36.3%), ‘professional competence’ (30.2%), ‘personality’ (10.8%), ‘communication’ (6.3%), ‘practice organization’ (5.9%), ‘ethical and moral behavior’ (5.7%), and ‘I do not know, or I have no idea’ (4.9%). The public can help medical students to construct their professional identity by supporting the exploration of and commitment to professional values that society expects of physicians. Ideally, fusing medical expertise with social skills will fulfill the ideal of what the public considers a ‘good’ doctor. This shared definition of a ‘good physician’ has several implications for medical education. Future physicians can benefit from education about the general population’s medical needs as well as personal needs, fears, and concerns.

## Introduction

Becoming a medical professional is not only about the accumulation of medical knowledge and skills, but also about core values and essential elements like ethical principles and communication skills; it is also about the acquisition of a new identity – an identity as a physician [1–3]. Professional identity formation (PIF) is a multifaceted development consistent with the competencies and values of the medical profession, intended by both medical students and educators, perceived by them and by the public or their future patients [3]. The goal of identity formation is to transform medical students into physicians and prepare them for the needs of the community and society [4].

Medical education characterizes PIF as a dual process: at the individual level, which involves psychological development; and at the collective level, which includes role socialization and participation in the community’s work [5]. Until now, medical education has focused on socialization, specifically the influence of experienced professional role models [6], participation in a community or communities of practice [7], and clinical encounters with patients as factors in PIF [8,9].

Bleakley and Bligh suggest relocating physicians’ identity construction away from identification with senior physicians as role models to an authentic patient-centered model, ‘where sustained early patient contact offers a basis for accelerating the forming of tacit knowledge (scripts, pattern recognition, and encapsulated knowledge) as the basis to clinical expertise’ [10]. A person becomes a physician in relation to others: patients, colleagues, and public members. Therefore, roles are external characterizations defined by others. Clinical and non-clinical experiences also impact the development of a learner’s medical professional identity through conscious and unconscious pathways [11]. Understanding this process in a way that supports and promotes this identity shift is critical in preparing physicians to work adaptively in evolving systems of care, take advantage of new technologies, and meet changing health care needs [12].

Experience gained from direct encounters with patients and other public members is foundational to a physician’s identity [5,9,13]. However, the critical role that patients or the public can play in PIF outside the clinical learning environment has received little attention. Nevertheless, active patient engagement should become an increasingly central component of education to help students explore their role as health professionals [14,15]. Active public and patient involvement (PPI) is an essential part of quality assessment and reporting, priority setting, clinical practice guideline development, and implementation, health technology, comparative effectiveness research, and health governance [16–19]. In the last few years, PPI increasingly encompassed student selection and admission, curriculum development, course management, faculty development, and program evaluation [18,20]. Therefore, medical professionalism, as seen by the public, should be more central to medical care [15]. It should be a priority in professional life, practice, education, regulation, and research to achieve good medical practice for everybody.

As of now, more knowledge is required to develop students’ medical professional identity through comprehensive curricula. However, to take this step, it is necessary to determine which elements and behaviors are associated with the concept of medical professionalism. The way in which societies talk about ‘ideal doctors’ shapes how medical educators and students understand and implement the process of becoming one [21]. The discussion of identity formation is underpinned by the widespread assumption that there is an ideal ‘good doctor’ identity that students and trainees are taught and must grow into [22]. The ‘ideal doctor’ can be perceived differently by the nursing staff [23,24], practicing doctors [25–28], medical students [2,29–31], the public [25,32,33], or patients [24,26,27]. However, there is limited knowledge about the Austrian public’s perspective on the concept of an ‘ideal’ or ‘good’ doctor.

To look beyond professionalism as a measurable competency, educators have emphasized the importance of forming a professional identity in which learners ‘think, act, and feel like doctors’ [3–6,34]. None of the studies addressed professional identity as perceived by the public. Since identity cannot be observed, we expeced descriptions of behaviors as indicators of an underlying identity structure.

Furthermore, in this study, we assumed that the public views and assesses professional behavior from a different perspective than the medical staff. As we are striving to strengthen the responsiveness to the needs and expectations of the public, we used the term public instead of patient to include people with health problems and healthy people, community members, and laypeople. In doing so, we sought to bridge the gap between ‘knowing how to act as a medical professional’ and ‘acting as a medical professional so that everyone can perceive this professional medical identity’.

### Aim

This study aimed to examine the public’s perception of doctors’ ideal qualities by analyzing their representation of both ‘good’ and ‘bad’ doctors. Beyond physicians’ particular characteristics, we also investigated whether the ‘bad’ doctor can be defined as an extension or a contrast to the image of a ‘good’ one. Therefore, we collected statements from the public about these characteristics. We categorized the statements to obtain a comprehensive description of medical professional identity.

## Method

### Study design

In this study, we used a qualitative approach with an open-ended questionnaire. The questions were as follows: ‘In your opinion, what is a good doctor? In addition, what else do you think makes a good doctor? How would you describe him or her?’ and ‘In your opinion, what is a bad doctor? In addition, what else do you think makes a bad doctor? How would you describe him or her?’ The answers were categorized via content analysis by a psychologist and a physician (JSG and AH).

### Data collection

Data were collected through an anonymous, nationwide computer-assisted telephone interview (CATI) with 1000 Austrian citizens. An experienced research institute (Austrian GALLUP Institute) conducted the interviews between February and March 2020. For this purpose, random telephone numbers were generated using the random last digit (RLD) dialing method, which ensures that people not listed in the telephone book are included in the sample. For this study, 80000 randomly generated telephone numbers were available, with 70% mobile numbers and 30% landline numbers. Table 1 shows the dropouts proportional to the interviews. The CATI system sorts the numbers to control the proportion of mobile and landline numbers.

**Table 1.** Sampling distribution characteristics; 2020 good doctor survey.

Sampling distribution	n
Numbers used	66872
Completed interviews	1000
Refusal or drop out	5795
Answering machine/no one picks up	32418
Invalid telephone numbers	26441
Language problems	231
Not available (e.g., sick or on holiday)	590
No private household	397

To ensure representativeness, a quota sample was obtained for gender, age, federal state, level of education, and city size. The criteria for representativeness were a sufficiently high number of cases, comparatively small ranges of variation of ± 1.4 to ± 3.2 for a sample of n = 1000 interviews, simple random sampling, and each person had the same chance of becoming part of the sampling.

Exclusion criteria were no consent, unwillingness to participate, or difficulties with the German language that hindered them from understanding or answering the questions. Informed consent was obtained from all participants. The questions had been pretested on a small sample (N = 20). Preliminary data were not included in the subsequent analysis.

The interviews were conducted in German and had an average length of 14 minutes.

### Data analysis

Interviews were recorded, transcribed verbatim into electronic form, and anonymized. We subsequently analyzed the transcripts with MAXQDA 2020 (Verbi GmbH) using Mayring’s content analysis, a systematic qualitative method for identifying, analyzing, and reporting patterns and themes within data [35].

All responses were grouped into thematic categories. A list of key categories taken from Luthy et al. [36] was used as a template to identify and categorize the responses.

One researcher (JSG) analyzed the transcripts and iteratively developed categories. Simultaneously, a second researcher (AH) interpreted approximately 20% of the material. The coding structure and the emerging conceptual framework were iteratively developed and critically discussed with two more researchers (VSH and HK) until a consensus was reached. Finally, the whole material was re-worked by JSG according to the accepted coding scheme.

### Participants

Among the 1000 participants, 51.5% were women and 48.5% were men. Participants had the opportunity to choose which of the following four categories they assigned themselves to female, male, diverse, or I do not want to categorize my gender. On average, female respondents were 48 years (SD = 15.46) and male respondents were 45 years old (SD = 16.07). Age ranged from 18 to 75 years (M = 46.4; SD = 15.8) (Table 2).

**Table 2.** Sample selected characteristics (n = 1,000); 2020 good doctor survey.

Characteristic	Participants (n, %)
*Sex*	
Female	515 (51.5)
Male	485 (48.5)
*Age Group (years)*	
18–30	215 (21.5)
31–40	168 (16.8)
41–50	188 (18.8)
51–60	215 (21.5)
61–75	214 (21.4)
*Country of Birth*	
Austria	963 (96.3)
Other	37 (3.7)

### Translation

Translation of the codes and statements into English was based on international standards and principles of good practice for the translation and cultural adaptation [37]. The first author translated the statements into English, taking care to preserve the original meaning. Two colleagues whose native language is German then independently translated this first version backward into German. Translation discrepancies were discussed until a consensus was reached. The retranslation was then compared with the original German-language version, revealing minimal differences, which could be clarified via communicative validation. Then, an English language editing service professionally edited them.

### Ethical considerations

After explaining the study objectives, participants gave their consent for interview and recording. Confidentiality was guaranteed and all responses were anonymized. Participants had the right not to answer questions and could withdraw from the study. As no clinical trial was performed and patients were not involved in this study, the ethical committee of the Medical University of Vienna granted an exemption from the ethics approval requirements. The study protocol was in line with the ethical guidelines of the Declaration of Helsinki on Good Clinical Research Practice.

## Results

**Figure 1.**
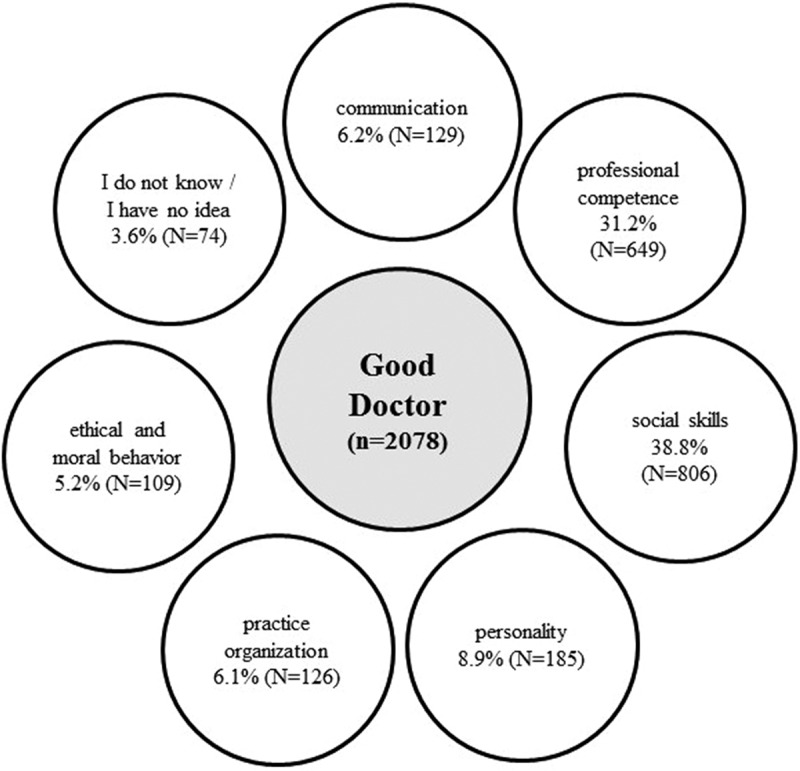
Categories for ‘good’ doctors by 1000 Austrians: distribution of answers through the seven main categories.

**Figure 2.**
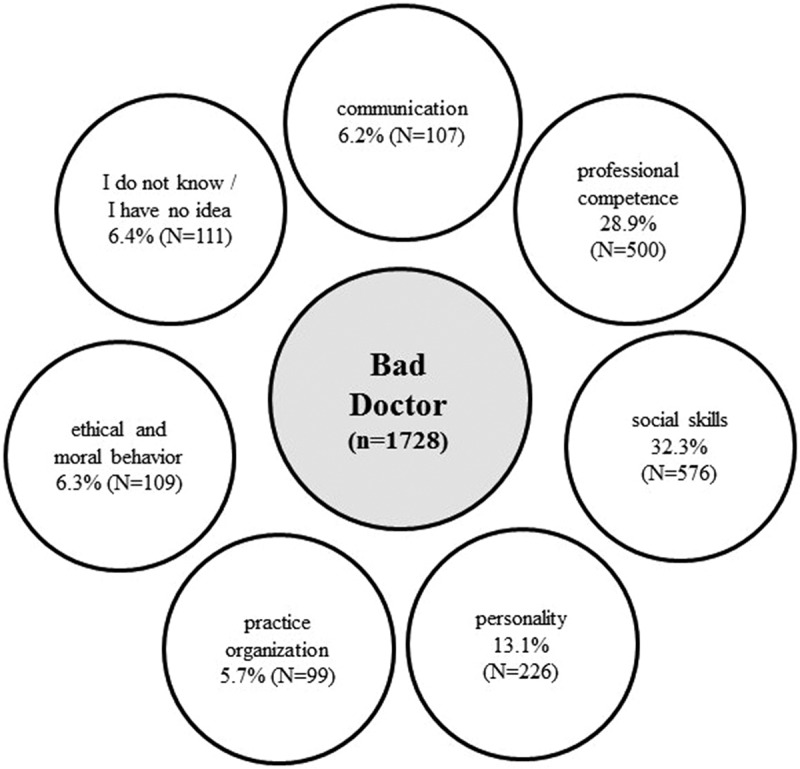
Categories for ‘bad’ doctors by 1000 Austrians: distribution of answers through the seven main categories.

Overall, 1000 participants gave 3806 single responses. We collected, compared, and coded 2078 answers for ‘good’ and 1728 for ‘bad’ doctors. Based on the statements, the content analysis distinguished seven main categories, with some degree of overlap between them: ‘personality’, ‘social skills’, ‘communication’, ‘professional competence’, ‘practice organization’, ‘ethical and moral behavior’, and ‘I do not know, or I have no idea’. Figures 1 and 2 are summarizing the responses for the main categories the public considers a ‘good’ and a ‘bad’ doctor. Table 3 shows the seven main categories and the considerations how statements were assigned to which category.

**Table 3.** Definition of the categories.

Categories	Definition
Personality	Personality traits are personal factors that push people toward or away from professional behavior. Personality refers to characteristics of the person that are relatively stable and consistent. The focus is on general personality traits such as ‘being nice’ and ‘polite’.
Social skills	Social skills are dispositions of physical and psychological action; these are action-centered. The difference between social skills and the personality of a good doctor is that skills are about peak performance, whereas personality is about typical behavior. Therefore, this category contains specific behaviors, such as ‘listening’, and ‘responding empathically to patients’.
Communication	During medical treatment or consultation, communication is one of the central tasks of doctors. They have to listen carefully, understand the needs of their patients, or have them explained to them. At the same time, doctors must be able to explain medical issues adequately themselves. It includes patient-friendly language, answering questions, open communication, and explanations about treatment or medication.
Professional competence	Professional competence is at the heart of medical practice. Therefore, this includes specialist knowledge, medical skills, and abilities of diagnosis, examination, treatment, healing of diseases, and pain relief. It also contains alternative medical knowledge as a counterpart to academic medicine.
Practice organization	This category includes all tasks not directly related to medical treatment. Doctors manage teams and surgeries; they offer services, such as opening hours, home visits, and night services.
Ethical and moral behavior	The relationship between patient and doctor requires trust and honesty. Therefore, this category includes integrity, confidentiality, and independence from pharmaceutical companies, motivation, and passion for the profession as a doctor. The ability to self-reflect and recognize one’s own limits are central. At the same time, this category includes respect for and consideration of population groups, e.g., age, gender, religion, and culture.
I do not know or I have no idea	Some interviewees indicated that they could not or would not answer the interview questions. Some stated that they did not feel confident to make a judgment or did not have an answer.

### Attributes of ‘good’ doctors

‘Social skills’ is the largest category and covers 38.6% of all ‘good’ doctor responses with 806 statements (Table 4). The answers mainly refer to doctors taking time for consultation and listening carefully to their patients. Doctors who are responsive to complaints, take care, are reliable and dedicated, and interact well are considered ‘good’. Statements referring to doctors that are understanding, attentive, helpful, reassuring, and motivating define this category. Respondents mention that doctors should empathize with patients’ medical problems and their situations.

**Table 4.** Main categories and subthemes of ‘social skills’ for a good doctor.

Main categories for ‘social skills’	nb of responses, n (%)	Subthemes	*Exemplar quotes*
Takes time	266 (33)		‘Takes time for the patients and doesn’t switch from one person to another every 2 minutes’; ‘takes enough time for a consultation’;
Listens	169 (21)		‘If one can listen to what is wrong with the patient’; ‘Someone who listens to me about problems’; ‘Someone who can listen well’;
Taking care	126 (15.6)	To be responsive to complaints Takes care Reliable dedicated Good interaction	‘Should respond to the individual needs of the patient’; ‘Has sympathy for the concerns of his patients’/ ‘Someone who takes care of you – for a long time’; ‘He or she should take care of patients’; ‘Takes good care of patients’; ‘Dedicates himself with full passion to the patients’ / ‘If I can rely on him’; ‘Is reliable’; ‘One who intercedes on behalf of patients’ / ‘Someone who is good with patients’; ‘Good interactions with patients’;
Respond to the individual	98 (12.2)	Understanding Personal support A partner for health Attentive Helpful Reassuring Feels in good hands Patients are human beings Considerate – respectful Proactive thinking Motivating	‘Understanding of the social background of individual patients’; ‘Who understands the patient’s opinion’; ‘Understanding for my problems’/ ‘Individual support’; ‘Personal care’; ‘Supports well’;/ ‘Partner in health matters’; ‘A partner for health’; ‘Based on partnership’/ Attention to the patient’; ‘be attentive’; / ‘If he is helpful’; ‘If he is willing to help’/ ‘Is reassuring before unpleasant situations during the exam’; ‘Can take away the fear of an examination’;/ ‘When you feel in good hands in the practice’; ‘One feels in good hands’/ ‘Always keeps in mind that his patients are human beings’; ‘You get the feeling that you are a human being there’; ‘Does not see patients only as objects’/ ‘Considerate’; ‘Be prudent’; ‘Respectful interaction also with my wife’; ‘Shows respect’/ ‘Proactive thinking’/ ‘Can motivate patients’; ‘Health for motivation’; ‘Motivating’;
Empathic	69 (8.6)		‘Empathy for my medical problems’; ‘Can put himself in his patient’s shoes’; ‘Being able to empathize well with my situation’ ‘A good knowledge of human nature’; ‘Have an understanding of human nature’; ‘Someone who has a feeling for people’;
Takes someone seriously	33 (4.1)		‘If I have the feeling that I am not being taken seriously, if I am afraid of an illness or if a painful therapy is imminent’; ‘Takes the patient’s complaints seriously’; ‘Who takes the patient’s feeling seriously’;
Interested	28 (3.5)	Being there for someone She or he knows me	‘Is simply interested in how his patients are doing’; ‘Who is interested in his patients’; / ‘Someone who is there when you need him’; ‘He should be there for the people’; ‘Who is there for me’/ ‘Knows his patients’; ‘Who knows me well’;
Social skills	8 (1)		‘Have social skills when dealing with people’; ‘Social skills’; ‘Social competence’;
Negative characteristics to avoid	5 (0.6)		‘Does not seem overstrained or stressed’; ‘Not hectic’; ‘Is not too stressed’

With 649 statements, the category ‘professional competence’ comprises 31.1% of all ‘good’ doctor responses and is thus the second most frequent category (Table 5). The answers on medical competence mainly refer to proper diagnostic and therapeutic skills, correct, accurate, fast, and efficient diagnostics, and precise and thorough examination. The respondents also emphasize medical competence, flawlessness, and practical skills in their statements. This category includes professional education and training, extensive knowledge, and experience. Some mentions describe ‘good’ doctors as those who help with recovery, conduct correct and quick treatments and therapies, and treat patients well and painlessly. According to the public, on the one hand, doctors should prescribe correct medication fast, but on the other hand, they should not immediately and not only prescribe hard drugs. Further statements correspond to alternative or holistic medicine and the willingness to refer to other doctors.

**Table 5.** Main categories and subthemes of ‘professional competence’ for a good doctor.

Main categories for ‘professional competence’	nb of responses, n (%)	Subthemes	*Exemplar quotes*
Proper diagnostic/therapeutic skills	194 (29.9)	Correct and accurate diagnosis Precise, thorough examination Considers the patient’s circumstances Rapid and efficient diagnosis Searches for the cause of the disease	‘That he knows uncomplicatedly what the problem of my illness is and can determine that uncomplicatedly’; ‘good diagnostics and examination methods’; ‘he is supposed to be a good diagnostician’;/‘A good doctor makes the right diagnosis’; ‘makes the right diagnosis right away’;/‘Who performs a proper examination, even in case of flu’; ‘Thorough examination if necessary’; ‘someone who makes precise examinations’;/‘Involves all circumstances of the patient’s life in diagnosis (diet, exercise, work, etc.)’; ‘Involves the patient’s work and private environment in the diagnosis and treatment’;/‘When a diagnosis can be made quickly and efficiently’; ‘Good ability to recognize and heal a disease quickly’;/‘looking for the origin of diseases’;
Medical competence	172 (26.5)	Professional competence Flawlessness Practical skills	‘That they are competent and know their stuff’; ‘competent in medicine is important to me’; ‘have a medical competence’;/‘someone who has a good technical knowledge and can apply it well’;/‘Does not make mistakes’;/‘medical, practical skills’;
Expert knowledge	110 (16.9)	Professional education and training Good, extensive knowledge Has operating experience	‘can score points with specialist knowledge’;‘someone who understands his field of expertise’; ‘Knows well about his field of expertise’;/‘is always up-to-date with the latest medical developments’; ‘someone who is professionally well-trained’; ‘is at the cutting edge of science’;/‘Has extensive knowledge’;/‘good experience’; ‘good routine’;
General treatment	58 (8.9)	Helps with recovery Correct and quick treatment and therapy Treats me well Painless treatment	‘That she can help me and my wife when there are health problems’; ‘When the doctor tries to help the patient recover in the best possible way’; ‘someone who can help me when I am sick’/‘Who can help me quickly when I am sick’; ‘Effective and quick, acts without delay’; ‘treats not only symptoms but also factors of disease development’;/‘Treats people well’;/‘When it does not hurt to draw blood and vaccinate’; ‘Examination without pain and fear’;
Medication	46 (7.1)	Does not only prescribe medication Correct medication Does not immediately prescribe hard medicines Quick medication and prescription	‘not only prescribes medication but also considers other options’; ‘who is not only concerned with prescribing medication’; ‘who has alternatives to medication’; ‘prescribes as little medication as possible’;/‘asks the patient what medicines they have at home and prescribes them if needed’; ‘prescribes the right medication’;/‘does not immediately get any medication like broad-spectrum antibiotic’; ‘Does not prescribe the strongest medications right away’; ‘does not prescribe pills right away, rather thinks beforehand’;/‘Prescribes what I need in an uncomplicated way’; ‘When you get a prescription quickly’;
Offers holistic and alternative medicine	36 (5.5)		‘treats not only scientifically but quite alternatively’; ‘who shows alternatives to treatment, instead of only academic medicine’; ‘can heal on a spiritual level, e.g., acupuncture, without chemicals’; ‘who also prescribes alternative medicines, e.g., homeopathy’;
Referral to other doctors	15 (2.3)		‘All options and some basic knowledge to refer to the right specialists’; ‘who has enough connections to specialists’; ‘refers me when he does not know what to do’;
Specific doctor image	11 (1.7)		‘Private doctor, not from the regional health insurance fund’; ‘I would rather have a female doctor than a male doctor’; ‘Prefer male doctor’; ‘young doctor’; ‘general practitioner’;

The ‘personality of a good doctor’ consists of 185 ‘good’ doctor quotes and thus comprises 8.9% of all statements about ‘good’ doctors. It contains personal traits, such as being kind, patient, open, honest, polite, likable, and humorous. The statements refer to doctors who are humane, fond of children, and conscientious. To be a ‘good’ doctor, according to some, it is also necessary to avoid negative personality traits, such as being a snob or being ‘God in white’ (Table 6).

**Table 6.** Main categories of ‘personality’ for a good doctor.

Main categories for ‘personality’	nb of responses, n (%)	*Exemplar quotes*
kind – nice	61 (33)	‘Deals with every patient in a friendly manner’; ‘Friendly competent manner’; ‘Friendly charisma’; ‘Nice to all no matter young or old, educated or not’; ‘Is nice’; ‘Neatness’; ‘He should be friendly’;
patient – calm	31 (16.8)	‘Devote themselves with patience to the patients’; ‘Shows patience’; ‘Has patience for the patient’; ‘Radiating restfulness’; ‘Restful and understanding demeanor’; ‘Calm charisma’;
humanity	24 (13)	‘Humanly outstanding’; ‘Humanity in social matters’; ‘Should be human’; ‘Humanly, simply humanly’;
open – honest	19 (10.3)	‘Is open to the patient’; ‘Has an open manner’; ‘He is supposed to be honest’; ‘Is honest’; ‘Honest’;
polite	10 (5.4)	‘If he is polite’; ‘Politeness’;
likable	9 (4.9)	‘Be sympathetic’; ‘That the first impression is good’; ‘Chemistry must fit’;
precise	8 (4.3)	‘Should try to get a precise picture of what the patient wants’; ‘He should be precise’; ‘Precise work’
negative characteristics to avoid	6 (3.2)	‘Is not God in white’; ‘Not a snob’; ‘Not too strict’; ‘Not complicated’; ‘Not to be preachy’;
fond of children	6 (3.2)	‘Be fond of children’; ‘Is kind to children’;
conscientious	4 (2.2)	‘Be conscientious’; ‘Is conscientious’;
doctors’ personality	2 (1.1)	‘Is suitable for the medical profession in terms of personality’; ‘Depends on the personality’;
humorous	2 (1.1)	‘Humorous’; ‘Has humor’

With 129 statements, ‘communication’ covers 6.2% of all ‘good’ doctor responses and is, therefore, the fourth-largest category. The main topics in this category are comprehensive explanations with simple conversations and outlooks on treatment possibilities. It includes questioning and answering honestly, openly, and in a way, patients can understand. The interviewees emphasize that communication skills can create a friendly conversational atmosphere. Some statements highlight good, competent, and personal advice (Table 7).

**Table 7.** Main categories and subthemes of ‘communication’ for a good doctor.

Main categories for ‘communication’	nb of responses, n (%)	Subthemes	Exemplar quotes
Comprehensible explanations	48 (37.2)	Simple conversation Outlook on treatment possibilities Tells the diagnosis immediately	‘Be able to explain complicated issues simply’; ‘Understandable explanations of the clinical picture and medicines’; ‘Can understandably explain complex medical matters’;/‘Someone you can talk to on a normal level and not throw around Latin words’;/‘Someone who conveys a positive image when talking about the diagnosis’;/‘explains well what disease you have, understandable for laypeople’;
Communicative	38 (29.5)	Good communication skills Friendly, good atmosphere for conversation	‘Someone with whom you can also talk confidentially at eye level’; ‘detailed conversation about the reason for my visit’; ‘also talk about nonmedical topics’/‘that there is a good communication’; ‘bring along communication skills’/‘trustful and nice conversation like with a good friend’; ‘Trusting and warm basis for conversation’;
Advice	24 (18.6)	Good, competent, personal advice Gives tips and advice	‘Good advice when I travel abroad, regarding vaccinations and prophylaxis’; ‘Can advise me well’; ‘personal advice’/‘Advises for healthier living’; ‘Advises on better health’; ‘Tips about preventive care’;
Questioning and answering	19 (14.7)	Answers questions Asks questions	‘Responds to the patient’s questions’; ‘Who patiently answers the questions’; ‘Gives useful answers’;/‘Ask about diagnostic findings’; ‘asks questions where necessary’;

With 126 individual statements, the category ‘practice organization’ comprises 6% of the ‘good’ doctor. According to the respondents, doctors should be available, reachable, and decisive. This category includes special services such as good opening hours, house calls, and night duties (Table 8).

**Table 8.** Main categories and subthemes of ‘practice organization’ for a good doctor.

Main categories for ‘practice organization’	nb of responses, n (%)	Subthemes	*Exemplar quotes*
Reachability	86 (68.3)	Quick appointments Good opening hours	‘Easy to reach when you need an appointment’; ‘someone easy to reach’;/‘gives appointments, no long waiting times for appointments’;/‘has long opening hours’;
Practice	23 (23)	Equipment of the practice Local practice Good team	‘Modern and well-equipped practice at the cutting edge’; ‘modern equipped practice rooms’; ‘well-equipped practice’;/‘located near me’; ‘Is not far away’;/‘Good team’; ‘teamwork’;
Availability	17 (13.5)	House calls and night duties Sick notes In-house pharmacy	‘Quick and uncomplicated home calls’; ‘Shall make home calls’; ‘night services’;/‘who writes me sick sometimes if I feel bad or had trouble with my girlfriend’; ‘writes me a sick note uncomplicated’;/‘an in-house pharmacy so that I can save myself the trip to the pharmacy’

‘Ethical and moral behavior’ consists of 109 ‘good’ doctor quotations, which accounts for 5.2% of all statements and is, therefore, the smallest category. This subject includes honesty, integrity, trustworthiness, confidentiality, motivation, and passion for the work beyond financial interests or obligations to the pharmaceutical industry (Table 9).

**Table 9.** Main categories and subthemes of ‘ethical and moral behavior’ for a good doctor.

Main categories for ‘ethical and moral behavior’	nb of responses, n (%)	Subthemes	*Exemplar quotes*
Morals	80 (73.4)	Trustworthy Doctor by passion and not for profit Role model No prejudices	‘A basis of trust that you can tell all your problems without taboos etc.’; ‘That I have the feeling that I can trust him’; ‘is trustworthy’;/‘does it not only for money and social position but for patients’; ‘practices the profession with passion’; ‘Is it for passion and not for profit’;/‘Is a role model’; ‘Is a fatherly friend’;/‘Does not have a prejudice against lesbian partnerships’; ‘without prejudices’;
Ethics	20 (18.3)	Discretion and confidentiality Treats all patients equally Independence from the pharmaceutical industry No experiments with medicines or therapies Adheres to his oath	‘That I don’t have to be afraid when I go to the doctor, and he passes on something’; ‘I can talk confidentially without it being passed on’; ‘Discreetly’;/‘who takes care of the patient, no matter what the patient’s origin, at any time of the day or night’; ‘Treats all patients equally’;/‘must do something good for people and not for pharmaceutical companies’; ‘does not let himself be driven by pharmaceutical companies’;/‘No experiments with patients, e.g., drugs, therapies’; ‘Does not experiment on patients’;/‘Adheres to the Hippocratic oath’;
Altruism	9 (8.3)	Has a heart for people Has the well-being of the patients in mind	‘who has a heart for people’; ‘Someone dear to one’s heart’; / ‘the well-being of the patients is in the foreground’; ‘who has the patient’s well-being in mind’; ‘Strives for the well-being of patients’;

### Attributes of ‘bad’ doctors

The largest category of the ‘bad’ doctor is ‘social skills’ with 576 statements (32.9% of ‘bad’ doctor responses). The answers mainly refer to doctors who do not take time for their patients and do not liste attentively. According to the public, social incompetence is due to an arrogant, condescending, preachy, or overly theoretical manner. Doctors who do not respond to the individual, cannot soothe their patients, lack understanding, do not have personal contact, or go into too much detail are perceived as unsuitable. If doctors are not on a par with their patients, do not make eye contact, or do not know or recognize them, their behavior is perceived as disinterest. Social incompetence also includes a lack of empathy or care and the feeling of not being taken seriously (Table 10).

**Table 10.** Main categories and subthemes of ‘social skills’ for a bad doctor.

Main categories for ‘social skills’	nb of responses, n (%)	Subthemes	*Exemplar quotes*
Has no time	187 (32.5)		‘the doctor does not take time’; ‘If you have the feeling that there is no time available’; ‘A doctor who has no time for me’; ‘that he or she does not take time’; ‘then doctor takes only a short time’;
Social incompetence	102 (17.7)	Arrogant/Condescending Super-teachy Prejudiced Too theoretical	‘talk down to people’; ‘he must not be conceited’; ‘if he is arrogant’; ‘to be condescending’ / ‘To be super-teachy and wants to improve you’; ‘has a teacher-like demeanor’; ‘teacher-like’; / ‘someone who is not willing to think outside the box’; ‘a preconceived opinion’; ‘prejudiced thinking’/ ‘too theoretical’; ‘out of touch with reality’; ‘too factual’; ‘unworldly’; ‘abstract’;
Does not listen	69 (12)		‘When a doctor cannot listen to patients’; ‘if he/she cannot or will not listen’; ‘does not listen to the patient’; ‘Only listen superficially’; ‘cannot listen well’; ‘if he/she does not have an open ear’;
Is not responsive to the individual	64 (11.1)	Does not go into detail Has no understanding Not soothing No personal contact Does not suit me	‘no attention is paid to the specific problems of the patient’; ‘does not go into the patient’s complaints’/ ‘pretending to understand you, but in reality, it comes across differently’; ‘shows no understanding’; / ‘Does not spread panic in times like these, debunk media scaremongering’; ‘scares unnecessarily’; / ‘no individual support’; ‘no personal contact’; ‘no personal contact with the patient’; / ‘to personally assess whether he suits me or not’; ‘is personally unsuitable’;
Disinterest	63 (10.9)	Is not on a par with patients No eye contact Does not know patients Does not recognize patients	‘When no interest is shown in people’s symptoms and problems’; ‘When he is not interested in patients’;/‘My doctor must be able to communicate on a par’; ‘is not at eye level’;/‘only stares at computers instead of looking at the patient’; ‘when he does not look at me in conversation’; ‘no eye contact’;/‘if he does not know you’; ‘Confuses patients during surgery’;/‘where you don’t really feel noticed’; ‘does not recognize’; ‘does not deal with the patients’;
Does not take seriously	52 (9)		‘If one gets the feeling not to be taken seriously concerning the complaints because of which one has made an appointment’; ‘When you think the other person does not take you seriously or thinks you are faking something.’; ‘when the doctor does not take the patient’s statements seriously’;
Not empathic	33 (5.7)		‘He has no empathy’; ‘Is unable to put himself in the patient’s shoes’; ‘cannot empathize with patients’; ‘lacks empathy’: ‘not empathic’; ‘empathy is missing’;
No care	4 (0.3)		‘if you do not feel well taken care of’; ‘not in good hands’; ‘one should not feel left out’;

‘Professional competence’ covers 28.5% of all ‘bad’ doctor responses with 500 statements and is the second most frequent category (Table 11). Respondents emphasize poor, inaccurate diagnostic and therapeutic skills as well as superficial, unpleasant, or painful examinations. Frequently mentioned statements refer to poor, wrong, too strong, or too fast medication. Doctors are considered medically incompetent if they lack medical expertise or work too fast and make mistakes. The category also contains statements about mass processing and poor therapies, sloppiness, or symptom treatment. Some people also see the absence of alternative medicine, continuing education and training, or referral behavior as arguments for judging doctors as ‘bad’.

**Table 11.** Main categories and subthemes of ‘professional competence’ for a bad doctor.

Main categories for ‘professional competence’	nb of responses, n (%)	Subthemes	*Exemplar quotes*
Poor diagnostic and technical skills	118 (23.6)	Superficial, not thorough, inaccurate examination Too quick or self-provided conclusions Inaccurate diagnosis Uncomfortable or painful examination	‘Incorrect or inaccurate diagnosis’; ‘Does not make correct diagnoses’; ‘Poor diagnostician’;/ ‘Makes a diagnosis from 1 meter away’; ‘Who does not bother to examine’; ‘Only superficial examination is done’; ‘Makes a diagnosis without having examined the patient’; / ‘When I, as a patient, have to say what is wrong with me’; ‘Where you have to make the diagnosis yourself’; ‘A rash diagnosis is made’; ‘Making diagnoses too quickly’;/‘Who is uncertain about the diagnoses’; ‘Inaccurate diagnosis’; ‘Cannot make proper diagnoses’;/‘If you still have pain or more pain after treatment’; ‘Causes even more pain’; ‘Rough during examination’;
Poor medication	109 (21.8)	Only medication Too fast and too many medicaments Wrong medication Too strong medicines	‘A bad doctor prescribes only medicines’; ‘Who prescribes pills without proper diagnosis’;/ ‘When you have a problem, and he gives medication right away’; ‘When he prescribes too much medication too quickly’; ‘When unnecessary medication is prescribed’; ‘Too much chemistry’; / ‘When you get new drugs every time you visit again’; ‘Prescribes the wrong medication’; / ‘Prescribes medical bombs’; ‘Immediately starts antibiotics’; ‘Works with heavy drugs’;
Medical incompetence	85 (17)	No medical expertise Works too fast and makes mistakes	‘If professional competence is missing, he is a bad doctor’; ‘Not professionally good and competent’; ‘Does not master his art sufficiently’;/‘Without technical knowledge’; ‘Have no expertise’;/‘Is only fast’; ‘Works half-heartedly’; ‘Makes mistakes’;
Mass processing	77 (15.4)	Gets rid of patients short and quick Patients are just numbers	‘Only want to dispatch as many patients as possible as quickly as possible without examination’; ‘When there is mass processing’;/‘If he checks in quickly and immediately goes for the next patient’; ‘Where you are dealt with in fast-track procedure’;/‘Who treats patients as if they were a number’;
Poor therapy and treatment	53 (10.6)	Sloppiness Poor treatment Wrong treatment Only symptom treatment No treatment recommendation	‘He must not be sloppy’; ‘Who is forever meddling’; ‘Works sloppily’; ‘Sloppiness’; / ‘Poor advice and treatment’; ‘Treats patients poorly’; ‘Performs surgery poorly’;/ ‘Provably treats patients wrongly’; ‘Performs surgery, although it’s not necessary’;/ ‘Only symptom treatment’; ‘A doctor who treats the symptom and not the whole person’; / ‘He dismisses the patients again without any treatment recommendation, without them knowing what to do afterward’; ‘Is not caring about a sensible and feasible therapy’;
Lack of alternative medicine	27 (5.4)		‘Does not offer a medical alternative’; ‘Is completely against alternative medicine’; ‘No alternatives to medicines’; ‘Involves only academic medicine in healing’;
Lack of continuing education & training	16 (3.2)		‘Has no interest in continuing education’; ‘Is not up-to-date with the latest scientific findings’; ‘Has deficits concerning the state of research’; ‘Stops to educate further’; ‘Poor education’;
Referral behavior	12 (2.4)		‘Unnecessary referrals’; ‘When she does not send you to a specialist’; ‘When you are referred from one doctor to another’; ‘He should refer if he is unsure’;

‘Personality’ consists of 226 quotations, which accounts for 12.9% of ‘bad’ doctor statements. Most answers refer to impatient and stressed doctors. Negative personality traits are characterized by aloofness, unfriendliness, insecurity, overconfidence, rudeness, or superficiality (Table 12).

**Table 12.** Main categories of ‘personality’ for a bad doctor.

Categories for ‘personality’	nb of responses, n (%)	*Exemplar quotes*
Impatient/Stressed	89 (39.4)	‘when he has stress and transfers it to the patients’; ‘When he makes a stressed impression’; ‘Should not be impatient’; ‘The doctor is impatient’; ‘in stress all the time’; ‘stressed appearance’
Aloof	43 (19)	‘Being aloof, is God in white’; ‘appears aloof’; ‘Be aloof’
Unfriendly	35 (15.5)	‘Is unfriendly’; ‘Unfriendliness’;
Superficial	26 (11.5)	‘a superficial impression’; ‘be superficial’; ‘Too superficial’;
Rude	15 (6.6)	‘where you have the feeling that you are being waved off’; ‘when he is rude’; ‘extremely rude’; ‘dismissive’; ‘rude’;
Insecure	5 (2.2)	‘He should not be insecure’; ‘If he is so insecure’; ‘Insecurity’;
Lack of self-care	4 (1.8)	‘a cardiologist who is a chain smoker; I don’t take such a person seriously’; ‘a doctor who has a burnout after 30 years’;
Overconfidence	4 (1.8)	‘When he has overconfidence’; ‘self-opinionated’; ‘Overestimates himself’;
Not conscientious	2 (0.9)	‘Not acting conscientiously’; ‘Not acting in the patient’s best interest’;
Not fond of children	2 (0.9)	‘Is not fond of children’;

With 109 individual statements, the category ‘ethical and moral behavior’ comprises 6.2% of the ‘bad’ doctor. According to the respondents, a doctor’s behavior is perceived as immoral or unethical when it undermines integrity, trustworthiness, confidentiality, and assumed moral attitudes. The category contains the idea of a physician who is only interested in money or works for profit and not out of dedication. Undesirable physicians’ features, as indicated by some respondents, thus reduce their patients’ trust because they often have financial or other connections to pharmaceutical companies (Table 13).

**Table 13.** Main categories and subthemes of ‘ethical and moral behavior’ for a bad doctor.

Main categories for ‘ethical and moral behavior’	nb of responses, n (%)	Subthemes	*Exemplar quotes*
Immoral & unethical behavior	90 (82.6)	Financial focus Not trustworthy/not confidential Moral attitude Discriminating Dependence & influence from the pharmaceutical industry	‘Has become a doctor only because of money’; ‘Has designs on financial success and fame’; / ‘When he tells my daughter about my illness’; ‘Is violating medical confidentiality’; ‘One has no trust’; ‘Lack of trust’; ‘Is telling confidential things to others’; ‘Does not have trustworthiness’; / ‘My doctor is supposed to help me, not lecture me morally’; ‘No moral speeches’; ‘Moral lectures’; / ‘If he does not like foreign people’; If older people are treated differently (worse) than younger people because of their age; ‘Makes a difference in treating patients’; / ‘When people are not at the center of decisions, but rather profit or pharmaceutical companies’; ‘Dependent on pharmaceutical companies’; ‘No pharmaceutical servant’;
Self-interest	19 (17.4)	Not a philanthropist Not altruistic Practices only the profession Egoistic	‘Who does not care about humans’; ‘Misanthropic’; ‘Far from humankind’; ‘Not philanthropist’; / ‘Who does not care about the person’; ‘Does not care about the well-being of the patient’; / ‘Someone who rests on his laurels’; ‘When he is simply doing a job’; ‘Less idealism’; / ‘He is not even-handed’; ‘Selfish’

‘Communication’ covers 6.2% of all ‘bad’ doctor responses with 107 statements. The main topics in this category are incomprehensible and insufficient explanations, complicated and incomprehensible language, and unobjective comments. A physician, who does not speak, speaks too little, or initiates superficial conversation, creates a poor conversational atmosphere. Some interviewees highlight that poor communication is due to asking too many questions, not inquiring enough, or avoiding answers (Table 14).

**Table 14.** Main categories and subthemes of ‘communication’ for a bad doctor.

Main categories for ‘communication’	nb of responses, n (%)	Subthemes	*Exemplar quotes*
Incomprehensible, complicated language	45 (42.1)	Speaking or explaining in a complicated & incomprehensible way Insufficient or no explanations. Unobjective comments	‘Explains something in a complicated way, throw around torch vocabulary and in the end, it makes a complicated confusing impression on me’; ‘when technical vocabulary is used’; / ‘Without taking the time to explain’; ‘Does not explain sufficiently’; ‘Does not explain’; / ‘Unobjective attacks’; ‘Unobjective’;
Poor atmosphere for conversation	29 (27.1)	Does not speak or speaks too little Superficial conversation	‘When he speaks almost nothing, nothing at all’; ‘you cannot have a conversation with him’; ‘If he does not discuss everything in detail with the patient’; ‘Only superficial conversation’;
Poor question and answer behavior	23 (21.5)	Too many questions Does not inquire Does not answer questions.	‘Asks hundreds of complicated questions’; ‘Asks many questions’; ‘Superfluous questions’; ‘Who does not inquire about the patient’s condition’; ‘Without inquiring’; / ‘Ignores questions or answers them incomprehensibly’; ‘Who avoids my questions’;
Poor counselling & information	10 (9.3)		‘Someone who does not counsel the patients’; ‘Without objective counselling’; ‘Gives false information’; ‘Bad advice’; ‘Does not offer any information’; ‘Bad info’;

‘Practice organization’ consists of 99 quotations, which accounts for 5.6% of ‘bad’ doctor statements and is, therefore, the smallest category. This subject includes organizational deficits and poor accessibility, such as long waiting times for appointments in overcrowded waiting rooms (see Table 15). A few statements mention the equipment of the practice and its structure. According to the respondents, ‘bad’ doctors are not sufficiently available, have too many patients, and do not offer house calls or night duties.

**Table 15.** Main categories and subthemes of ‘practice organization’ for a bad doctor.

Main categories for ‘practice organization’	nb of responses, n (%)	Subthemes	*Exemplar quotes*
Poor accessibility	66 (66.7)	Waiting times for appointments Receptionist behavior Short opening hours	‘During calls, you only and always reach the audiotape’; ‘Poor accessibility’; ‘If he is not accessible’; / ‘Does not manage to prevent waiting times’; ‘Long waiting times for appointments and in the waiting room’; / ‘If only the receptionist finds time for a prescription without a conversation’; / ‘Strongly changing opening hours’; ‘Poor opening hours’; ‘Short surgery hours’;
No availability	18 (17.2)	Too many patients No house calls/night duties Work-to-rule No sick note	‘Does not look at the number of patients who come by in a day’; ‘Has too many patients’; / ‘Does not make house calls’; ‘He should come to our house at night when you need him urgently’; / ‘Works only according to the timetable’; ‘Stubbornly proceeds according to duty’; ‘Duty by the book’;/ ‘If she does not write me a sick note, even though I am unable to perform at the moment’; ‘Send me to work’;
Equipment of the practice	14 (14.1)	Poor structure Health insurance	‘Poor organization’; ‘Poor structure’; ‘Seems disorganized’; ‘Has no coordination’; / ‘Is bound to a certain health insurance’; ‘He costs something, so he is working in private practice’;

## Discussion

In our study, we investigated the characteristics of ‘good’ and ‘bad’ doctors to explore the public’s perception regarding the ideal qualities of physicians. Based on the analysis of the interviews, seven categories were identified. Most of the statements refer to either social skill or professional medical competence; these, therefore, seem to be valued qualities of good doctors.

When we look at the most frequent statements of the respondents, we discover the following definitions: An ideal physician could be defined as someone who takes plenty of time to listen attentively to the patients, can respond empathetically and sensitively to their concerns or complaints, and has medical expertise. By contrast, inadequate doctors have no time, do not listen attentively, appear impatient or stressed, treat their conversational partner arrogantly or condescendingly, and are medically incompetent.

The selected characteristics showed that the ‘bad’ doctor could be described at almost all times as the reverse image of the ‘good’ doctor. Inadequate doctors were more frequently characterized by their negative personality traits rather than their willingness or ability to communicate. The distinction between ‘good’ and ‘bad’ doctors is based on the capability to deal with patients and influence their behavior, and it depends more on skills such as attention, care, empathy, and interest than on medical expertise. Similar results have been obtained in various studies investigating perceptions of the public [19,23,33,38,39] or patients [36,40–45]. For example, Luthy and colleagues [36] evaluated patients’ perceptions of ‘good’ and ‘bad’ doctors; they used qualitative content analysis to extract eight characteristics of a ‘good’ doctor, namely scientific competence, sensitivity to emotions, positive personality traits, coping with each patient, availability, skillful communication, truthfulness, and lack of interest in financial aspects.

The objective of medical education among others is a developed professional identity of an ideal doctor. Achieving this goal requires more than excellent questioning, examining, diagnosing, and treating. Forming a professional identity needs more than operationalizing the sociological view of professionalism; it needs an internalization of professionalism through character development [34].

Our results underline the importance of teaching social skills, as aspects such as attentive communication and patient orientation require specific training to achieve peak performance [46]. The patient-physician relationship, communication, and social skills are essential for well-being and health [47–49]. These competencies should be acquired at the undergraduate level to provide a solid foundation for professional identity development [50].

The public has a conception of the ideal doctor within the health system, one who is equipped with the necessary human and professional qualities required for an optimal and effective doctor–patient relationship. This study can be taken as an indication that professional identity formation (PIF) in the context of medical education might be improved if the public perspectives were considered and used to inform and shape medical schools and curricula.

What is the best way to teach aspects like communication, personality, and social skills in terms of professionalism in medical schools? Reflection can be an important driver of personality change, and when we reflect on how we respond to new situations or unforeseen circumstances, this can lead to change [51]. The learning generalization model shows how personality can change through taking on roles such as ‘medical student’ [52]. Reflection might also help to raise awareness of institutional habits, challenge disempowering discourses, and legitimize identities [51]. Educators can promote this development through encouragement, provision of learning opportunities, and guided practice of principles and techniques [53].

Our data indicate that the public expects more focus on patient-centered values and interpersonal factors. Communication is a procedural skill that should be taught and trained, as this skill only improves with experience. It is crucial to educate and train real-life communication, such as active verbal, non-verbal and genuine listening. It refers to such things as eye contact, gestures, and body movement, but it can also include facial expressions, repetitive movements of the extremities, or vocalizations [54]. In most European countries, this has recently become an essential part of the medical curriculum [55]. However, there should be more guidelines for teaching social or communication skills.

It would also be conceivable to review the entire admission process, as medical educators often have no control over which individuals are admitted to the curricular process.

By adding a psychological development framework to character and behavior perspectives, we can better understand professional identity and professionalism and, more importantly, how the students themselves can influence the process of being able to think, act, and feel like a physician. Then, the professional identity formation moves from the hidden curriculum to the visible one.

### Limitations

The strongest aspect of this study is the inclusion of numerous respondents from different social backgrounds. Nevertheless, there are some limitations. First, our data were obtained using quota instead of random sampling. Not all elements of quota sampling are representative of the general population. Therefore, selection bias may have occurred, as there are only a few people with non-Austrian citizenship. As a result, some attitudes are likely to be over-represented. A second limitation is the translation bias. It might be possible that the translation from German into English is accompanied by a change in meaning.

Third, we did not try to make a difference between a ‘good’ and an ‘ideal’ doctor or between a ‘poor’ and a ‘bad’ doctor. ‘Poor’ doctors are seen generally as having good intentions but insufficient knowledge or skills for their job. However, ‘bad’ doctors, no matter how well-educated, trained, or qualified they may be, have bad, undesirable values and suspicious intentions. Characterizing someone as a ‘bad’ doctor implies moral deficiencies, even though these may coexist with laudable aspects of medical practice [32].

Our findings may adequately reflect the population’s views or their lay perceptions of ‘good’ medical care and treatment. Nevertheless, our research cannot be applied to all medical schools, medical students, or medical curricula indiscriminately. More comprehensive research would be needed before generalizations can be made.

## Conclusion

The involvement of the public in determining which attributes are necessary for good medical care is a positive way of ensuring the importance of such qualities, which combine clinical knowledge and skills with humanitarian values.

Active public involvement should be a central component of health profession education to help students explore their role as health professionals in collaborative, patient-centered practice, and shared decision-making.

Considering that perceived identities in medical education have an impact on the PIF, further research into the PIF process and the development of supporting curricula might be beneficial.

The authors believe that it would be reasonable to carry out further research in which students change their attributes, qualities, competencies, and values during training while knowing the needs and expectations of the public. 

If PIF is a focus of medical education, then engagement with professional values, moral concepts, ideas, and goals should be encouraged alongside integration into a community of practice. Community members as mentors could be invaluable allies in this substantial endeavor.

We hope that the public perceptions of ‘good’ and ‘bad’ doctors can help support medical educators’ efforts to support students’ active PIF processes and can be included in discussions leading to changes and developments within medical education programs.

## Data Availability

The datasets used during the current study are available from the corresponding author on reasonable request.

## Previous presentations

Part of this research were presented at the Annual Meeting of the Society for Medical Education (GMA 2021); September 16-17, 2021; Zurich, Switzerland
